# Allografts promote skeletal regeneration of periprosthetic femoral bone loss

**DOI:** 10.1016/j.jot.2025.04.004

**Published:** 2025-04-23

**Authors:** Simon von Kroge, Constantin Schmidt, Sebastian Butscheidt, Malte Ohlmeier, Michael Amling, Frank Timo Beil, Thorsten Gehrke, Klaus Püschel, Michael Hahn, Tim Rolvien

**Affiliations:** a Institute of Osteology and Biomechanics, University Medical Center Hamburg-Eppendorf, Lottestr. 59, 22529, Hamburg, Germany; b Department of Trauma and Orthopaedic Surgery, Division of Orthopaedics, University Medical Center Hamburg-Eppendorf, Martinistr. 52, 20246, Hamburg, Germany; c Department of Joint Surgery, Helios ENDO-Klinik, Holstenstr. 2, 22767, Hamburg, Germany; d Department of Legal Medicine, University Medical Center Hamburg-Eppendorf, Martinistr. 52, 20246, Hamburg, Germany

**Keywords:** Allograft, Bone regeneration, Bone transplantation, Osseointegration, Osteocyte, Revision arthroplasty

## Abstract

**Background:**

Periprosthetic bone loss is a common clinical problem in hip arthroplasty that must be addressed during revision surgery to achieve adequate implant stability. Although bone allografts represent the clinical standard among substitute materials used, evidence of their regenerative potential at the microstructural, cellular, and compositional level is lacking.

**Methods:**

A multiscale imaging approach comprising contact radiography, undecalcified histology, scanning electron microscopy, and nanoindentation was employed on human femoral explants obtained postmortem many years after allograft use during revision surgery.

**Results:**

The degree of skeletal regeneration through allograft incorporation between host bone and allograft bone was highly dependent on the defect depth (R^2^ = 0.94, *p <* 0.001), while no association between the allograft time *in situ* and incorporation (R^2^ = 0.06, *p* = 0.61) was apparent. The host bone-allograft interface showed a high overlap of 4.0 ± 2.9 mm and was characterized by active bone remodelling, as indicated by osteoid accumulation, high abundance of bone cells and vasculature. While bone cement generally limited the incorporation process, the osteocytic canalicular system of the host bone reached the allograft interface to guide bone remodelling.

**Conclusion:**

This is the first multiscale, histomorphometry-based evaluation of bone allografts used in revision hip arthroplasty for femoral bone loss in humans, demonstrating that they adequately facilitate skeletal regeneration through osteoconduction and subsequent remodelling.

**The translational potential of this article:**

This study identified the mechanisms and determinants of femoral defect regeneration through allografts on the basis of a unique sample collection. While our results support their favourable clinical outcomes, the scientific basis for incomplete incorporation is also demonstrated.

## Graphical abstract

**Figure:**
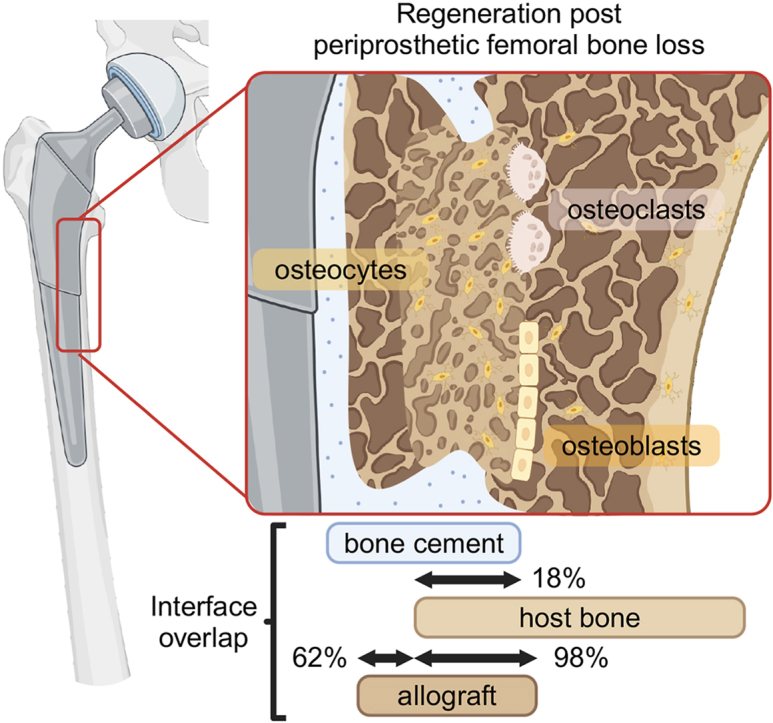

## 1 Introduction

Total hip arthroplasty is one of the most commonly performed surgical procedures worldwide, generally resulting in excellent outcomes [1,2]. Nonetheless, revision arthroplasty is often necessary due the occurrence of various complications, *e.g.*, aseptic loosening, periprosthetic fracture, or periprosthetic joint infection. A common side effect of these complications that must be addressed during revision is periprosthetic bone loss, which can occur in both the acetabular and femoral periprosthetic bone. In this context, bone allografts are frequently used to provide a stable basis for the fixation of the new implant [[3], [4], [5], [6]]. This procedure is generally successful in terms of clinical outcomes [7].

We have previously demonstrated through in-depth histological examination that both structural and chip allografts used during acetabular reconstruction are well incorporated by the host bone [8,9]. However, the regenerative capacity of allografts in the femur may vary, e.g., due to different loading patterns. Although few descriptive case reports indicated that allografts also inherit an osteoconductive potential in the case of femoral bone loss [[10], [11], [12]], an in-depth analysis has not yet been performed. Furthermore, although bone allografts represent the clinical standard among the substitute materials used, precise evidence of their regenerative potential at the microstructural, cellular and compositional level is lacking [13,14]. Not only factors stimulating the cellular process of allograft remodelling and thus revitalization, but also barriers limiting osteoconduction are unclear. It can be assumed that both fibrosis development and bone cement are limiting factors but the extent to which such hinder allograft incorporation is yet to be shown. Furthermore, whether osteoconduction or osteoinduction is the driving factor during incorporation is still to be allocated [14,15].

In this study, we therefore aimed to determine the incorporation properties of allografts used during revision arthroplasty for femoral bone loss, with a special focus on cellular and compositional mechanisms.

## 2 Materials & methods

### 2.1 Study cohort, sample acquisition and preparation

Patients undergoing femoral revision arthroplasty using structural and/or morselized allografts at one of the largest European centres for joint arthroplasty between 1987 and 2009 were included in a central registry. Clinical data, surgical reports, and perioperative images (*e.g.,* radiographs, Paprosky grade of periprosthetic bone loss [16]) were available for all patients. The study protocol is described in detail elsewhere [8,9]. In the following years, all decedents in the local crematory and the Department of Legal Medicine of the University Medical Center Hamburg-Eppendorf were matched with this registry [17]. In the case of a positive match, the consent of the relatives was obtained and the hips were explanted, fixed in 3.7 % formaldehyde, and further prepared.

**Fig. 1:**
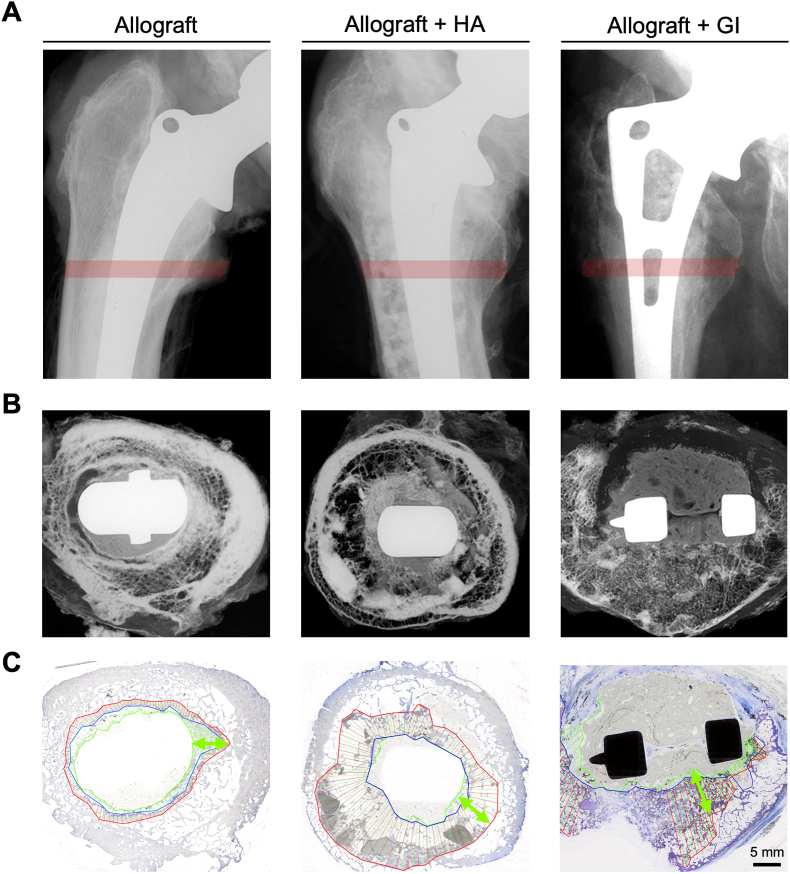
**Specimen acquisition and processing of femoral sections containing allograft bone. (A)** Contact radiographs of the proximal femur after revision arthroplasty with impacted allograft bone, and hydroxyapatite (HA) and glass ionomer cement (GI) in two individual cases were analysed. The red colouring indicates the region of the prepared cross-section for further analysis. **(B)** Contact radiographs of the determined cross sections containing implants and periprosthetic bone to identify and localize allografts and additional bone substitute material. **(C)** Incorporation was analysed on undecalcified histological sections of the whole cross-section stained with toluidine blue. Blue lines reflect the host bone border, red lines reflect the allograft bone border. Both lines imply the overlap (i.e., ingrowth). The green double arrow exemplifies the defect depth (i.e. the length between the cement interface and allograft bone border).

Specifically, to address the aims of this study, we obtained seven human whole femur explants with documented use of allografts during revision surgery to derive the incorporation *in situ*. The periprosthetic bone defects were all advanced, indicated by the high Paprosky grade (Table 1). A multi-method experimental design was chosen to determine all aspects of the incorporation processes. Surgical reports and clinical radiographs were used to identify the region within the femur where allograft bone was used. On digital contact radiographs, areas of the proximal femur containing allograft bone were readily identified and the height of the cross-sections selected for further analysis, which in all cases were at the level of the minor trochanter (i.e., metadiaphyseal) (Fig. 1A). Furthermore, two specimens in which synthetic bone substitute materials (BSM), namely glass ionomer (Ionogran; ESPE GmbH & Co KG, Seefeld, Germany) and hydroxyapatite, were used in addition to the allograft, enabled us to estimate the allograft-host bone borders more precisely (Fig. 1A). The areas of the host bone and allograft bone were identified in digital contact radiography of 5 mm thick cross-sections (Fig. 1B). These areas were subsequently analysed on undecalcified histological sections by determining the borders of allograft and host bone and specify their overlap to quantify the regenerative potential and incorporation of allograft bone on a bone-qualitative and cellular level (Fig. 1C).

**Table 1:** Overview of analysed explants obtained from individuals with allograft bone in the proximal femur postmortem.

Case no.	Age at death [yr.]	Sex	Age at bone graft [yr.]	Time *in situ* [yr.]	Graft material	Type of surgery	Paprosky grade
1	84	M	75	9	Chips + HA	4th revision (stem loosening)	IV
2	81	M	71	10	Structural + Chips	1st revision (cup and stem loosening)	IIIA
3	81	M	65	16	Chips + GI	1st revision (stem loosening)	IIIB
4	77	M	63	14	Chips	2nd revision (stem and cup loosening)	IV
5	86	M	73	13	Structural + Chips	1st revision (stem and cup loosening)	IIIB
6	70	F	55	15	Chips	1st revision (cup and stem loosening)	IV
7	95	M	80	15	Chips	1st revision (stem loosening)	IIIB

HA: Hydroxyapatite; GI: Glass ionomer.

Each femur was cut into two 5-mm thick cross-sections at the level where the largest amount of allograft was identified on clinical radiographs (i.e., the height of the minor trochanter). Subsequently, both sections were scanned using digital contact radiography (DCR, Faxitron, USA) to identify and localize allografts and additional BSM, followed by dehydration and embedding in plastic. From each specimen, one section was embedded in Technovit 7200 (Kulzer GmbH, Germany) for cross-sectional analysis to enable an analysis of the whole allograft bone-host bone interface (ground section). The second section was further cut into eight specimens, and embedded into methyl-methacrylate (MMA) for more detailed histological cellular characterization. In addition to the femoral specimens, iliac crest biopsies were acquired in all cases and compared with those of 95 age- and sex-matched control biopsies acquired in the context a previous study [18]. This study was approved by the local ethics committee under application WF-005/09 and complied with the Declaration of Helsinki.

### 2.2 Histology and histomorphometry

After dehydration and embedding of the whole cross-sections in plastic, histomorphometric analysis was performed on undecalcified toluidine blue stained 100 μm thick ground sections stained with toluidine blue. The other sections were cut in consecutive 4 μm thick cut sections and stained with toluidine blue and von Kossa as previously described [19]. On ground sections, the allograft bone border was defined as the most distant remaining non-vital allograft bone along the cross section, and the host bone border as the deepest ingrowth of host bone into the allograft. The overlap of both borders along the whole interface was defined as the host bone-allograft overlap. In addition to the ingrowth analyses, the extent of the femoral bone loss was characterized through determination of the bone defect area (i.e., the area between the cement interface and allograft border) and defect depth (i.e., the distance between the cement interface and allograft border). Furthermore, the fibrosis thickness and cement penetration were measured. Herein, the bone cement border was defined as the deepest penetration of cement particles towards host bone along the cross-section.

The local bone microstructure was analysed in von Kossa-stained sections. Specifically, the bone volume fraction (*BV/TV*, %), trabecular thickness (*Tb.Th*, μm), and trabecular number (*Tb.N*, mm^−1^) were captured in the allograft bone (AB), host bone (HB), and overlap region (Ov). To determine the remodelling activity, the proportion of unmineralized (*i.e.*, osteoid) bone and mineralized bone in terms of osteoid volume per bone volume (*OV/BV*, %) was determined. To evaluate the cellular activity in the AB, HB, and Ov, the percentage of bone surfaces covered with bone-forming osteoblasts (*Ob.S/BS*, %) and bone-resorbing osteoclasts (*Oc.S/BS*, %) were assessed. In addition to specific characteristics of bone quality and remodelling, the number of vascular channels (*N.VC/Ma.Ar*, mm^−2^) and the proportion of vascular channel area per marrow area (*VC.Ar/Ma.Ar*, %) were determined. Bone structural and osteoid indices were also evaluated in iliac crest biopsies. All parameters were determined according to the guidelines by the *American Society for Bone and Mineral Research* (ASBMR) [20].

### 2.3 Quantitative backscattered electron imaging

To determine the mineral density distribution of allografts and host bone, quantitative backscattered electron imaging (qBEI) was performed as previously described [21]. In brief, the embedded cross-sections were polished, carbon-coated, and scanned in a scanning electron microscope (LEO 435, LEO Microscopy Ltd., Cambridge, England) attached to a backscattered electron detector (Type 202, K.E. Developments Ltd., Cambridge, England). The generated grey-value images were evaluated individually in the host bone, overlap region, and allograft, by allocating grey values to the local calcium content. Thereby, the mean calcium content (*CaMean*, wt%), the peak calcium content (*CaPeak*, wt%), and the standard deviation of the calcium content distribution (*CaWidth*, wt%) was determined. In addition, the number of osteocyte lacunae per bone area (*N.Ot.Lc/B.Ar*, mm^−2^) and the osteocyte lacunar area (*Ot.Lc.Ar*, μm^2^) were analysed.

### 2.4 Electron dispersive X-ray spectroscopy

To specifically determine which elements were dissolved from the bone supplemental materials, whether components were incorporated within the bone matrix, and whether the surrounding bone matrix was impacted by the hydroxyapatite or glass ionomer, electron dispersive x-ray spectroscopy (EDX) was performed [22,23]. Polished cross-sections were analysed with a Crossbeam 340 (Zeiss, Germany) system assembled with an EDX detector (X-Max 80, Oxford Instruments, UK). Each region was imaged over a representative area of at least 1 mm^2^ and additionally at four points within these areas. A minimum of 4 million counts per measurement was used to get a representative amount of information. The imaging settings were set to a voltage of 30 kV, an acceleration current of 400 pA, and a 400× magnification.

### 2.5 Acid etching and scanning electron microscopy

To image the osteocyte lacuno-canalicular network, embedded samples were polished and acid etched based on a previously published protocol to resolve the upper layer of mineralized tissue and expose the resin-filled osteocyte lacunae and attached canaliculi [24]. After acid-etching, the samples were gold sputter-coated and imaged with a Crossbeam 340 (Zeiss, Jena, Germany) scanning electron microscopy attached with a SE2 detector. To image the osteocyte lacuno-canalicular network, a voltage of 3 kV, an acceleration current of 400 pA and a 1200× magnification was chosen.

### 2.6 Nanoindentation

Mechanical properties of the bone matrix in each region of interest were analysed by nanoindentation using an iMicro nanoindenter (KLA instruments, USA) equipped with a Berkovich diamond tip. Prior to testing all embedded specimens were ground to a co-planar finish and surface-polished. Allograft, host bone, overlap, and BSM of each sample were tested with 20 indentations at a distance of at least 20 μm between each indent. Using the integrated light microscope, the correct position and appearance of all indents was validated. Indentations were performed in depth-sensing continuous stiffness mode with a target depth of 5000 nm and a target indentation strain rate of 0.05 s^−1^. Calibration was carried out on fused silica before and after each measurement. Based on a previously described method and using the software provided by the manufacturer, the *Hardness* (GPa) and *Elastic Modulus* (GPa) were obtained applying a Poisson's ratio of 0.3 [25].

### 2.7 Statistical analysis

Statistical analysis was performed using SPSS software (v 24.0, IBM, USA). All data was analysed with the Kolmogorov–Smirnov test to test for normal distribution. Regional differences within each sample, *i.e.*, between the allograft bone (AB), host bone (HB), and overlap area (Ov), were determined by ANOVA and subsequent post hoc (Dunn) testing or by Kruskal–Wallis tests in case of non-parametric variables. Data is presented as mean ± standard deviation, while graphs specifically indicate specimens with additional bone substitute materials. Linear regression analyses were performed to determine associations between potential influencing factors and parameters of allograft incorporation. A significance level of 0.05 was chosen.

## 3 Results

### 3.1 The allograft-host bone overlap in the proximal femur is dependent on the defect depth

**Fig. 2:**
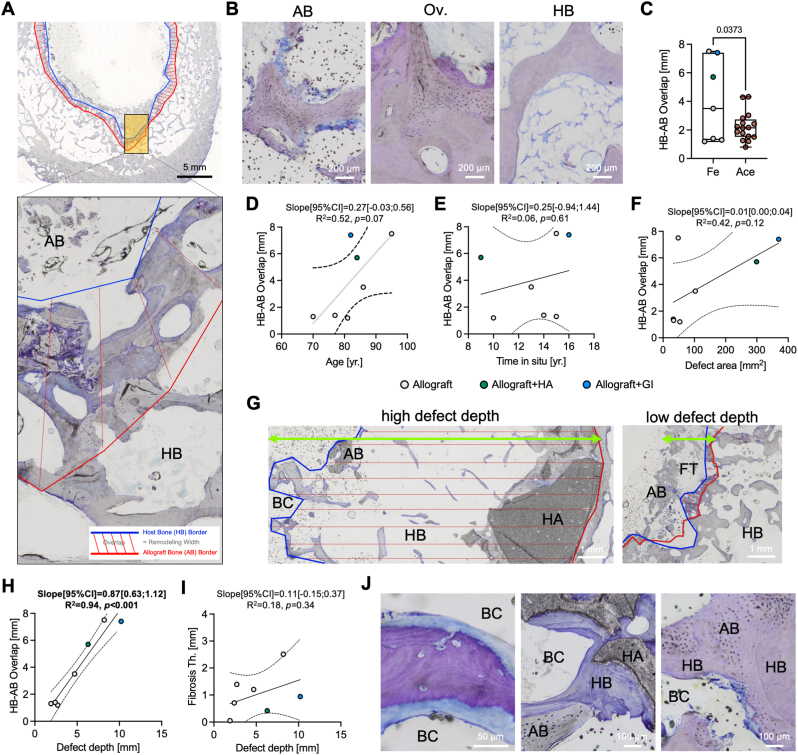
**Interface analysis of allograft and host bone indicates an association of defect depth and incorporation. (A)** At the interface of allograft (AB) and host bone (HB) an overlap comprising the fusion of viable and non-viable bone was detected. **(B)** Allograft bone presented with cell-free osteocyte lacunae and bone cement or fibrotic tissue surrounding bone fragments. The overlap region was characterized by a fusion of non-viable AB and viable host HB. Host bone presented with viable osteocytes and osteoblast as well as osteoclasts at the bone surface. **(C)** The mean overlap of HB and AB in the proximal femur (Fe) was higher than previously reported overlap in the acetabular (Ace) component. **(D**–**F)** Analysis of the associations of patient age, allograft time *in situ*, and defect area with overlap showed no significant results. **(G)** Representative illustrations of the overlap of HB and AB in cases with a high and low defect depth (green arrow). While a large overlap was found at high defect depth (left) with hydroxyapatite (HA), a low defect depth (right) was associated with a small overlap and fibrosis development. FT: fibrous tissue, BC: bone cement. (**H**) Strong significant association between the defect depth and overlap. **(I)** The defect depth was not associated with fibrosis thickness. **(J)** Left: Signs of demineralizing effects in the presence of bone cement, but also osteoconductive potential of allograft bone and additional hydroxyapatite or solely with allograft bone (right) in the presence of bone cement.

Allograft bone was detected histologically in all available specimens, enabling an in-depth characterization of its incorporation properties (Fig. 2A). Specifically, the remaining allograft bone, which was characterized by the absence of bone cells and empty, *i.e*., cell-free, osteocyte lacunae, was partly incorporated in bone cement and surrounded by fibrotic tissue (Fig. 2B, Figure S1). At the allograft-host bone interface, an overlap of both non-viable and viable regions was apparent. The host bone was primarily characterised by viable bone covered by osteoblasts and osteoclasts and filled with bone marrow (Fig. 2B–Table S1). In total, an overlap of host bone and allograft bone was present at 98.0 ± 3.7 % of the total interface with a mean overlap of 4.0 ± 2.9 mm (Table 2). In comparison to previously analysed acetabular explants, the overlap of host bone and allograft bone was higher (*p* = 0.0373) in femoral specimens (Fig. 2C). However, when excluding the cases with additional synthetic bone substitute materials, this difference was no longer apparent (*p*_*excl*_ = 0.323). At the interface sites where allograft bone or synthetic bone substitutes had no ingrowth of host bone, fibrous tissue was evident (Figure S1).

**Table 2:** Specification of the main determinants for femoral allograft incorporation including the interfaces of host bone (HB), allograft bone (AB), and bone cement (BC).

Parameter	Mean (SD)
AB-HB overlap of total interface [%]	98.0 (3.7)
Mean AB-HB overlap [mm]	4.0 (2.9)
Maximum AB-HB overlap [mm]	7.8 (4.8)
BC-HB interface of the cross-section [%]	44.0 (23.9)
BC-HB overlap of its interface [%]	18.2 (22.0)
Maximum BC-HB overlap [mm]	2.7 (3.0)
BC-AB interface of the cross-section [%]	62.3 (18.1)

Neither the patient age at revision surgery, allograft time *in situ*, nor defect area was associated with the mean host bone-allograft overlap (Fig. 2D–F). However, a higher ingrowth of host bone into the allograft was identified in deeper defects (Fig. 2G), indicated by a strong association between the defect depth and overlap (R^2^ = 0.94, *p* < 0.001) (Fig. 2H), while no association between the defect depth and fibrosis thickness was observed (Fig. 2I). Of note, no overlap of bone cement and host bone was apparent in three specimens. Bone cement in the other four specimens partly penetrated the host bone and reached the endocortical region. In these specimens, an overlap of bone cement and host bone was only apparent at 18.2 ± 22.0 % of its interface, which was present at 44.0 ± 23.9 % of the total cross-section, with a maximum bone cement-host bone overlap of 2.7 ± 3.0 mm (Table 2). Although bone cement generally presented as a limiting factor for allograft-host bone overlap, signs of bone formation and/or demineralization at its interface to host bone were apparent. Interestingly however, an osteoconductive capacity of allograft bone was also observed in the presence of bone cement, independent of additional synthetic hydroxyapatite (Fig. 2J).

### 3.2 High remodelling rate at the allograft-host bone interface

**Fig. 3:**
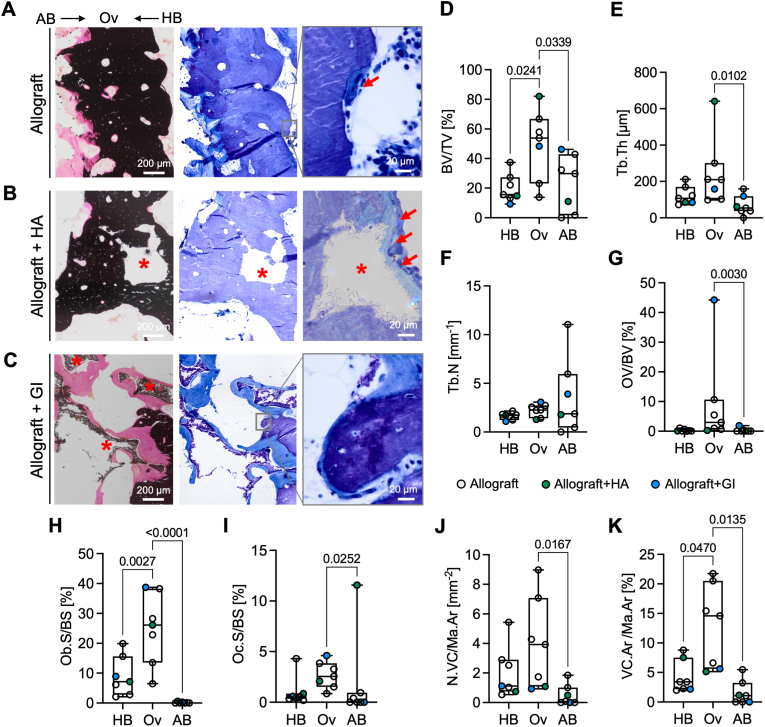
**The host bone-allograft bone overlap is characterized by fusion and high remodelling. (A)** Von Kossa- and toluidine blue stained sections were analysed indicating osteoid seams (pink) and bone cells, *i.e*., multi-nucleated bone resorbing osteoclasts (red arrow) and bone forming osteoblasts, at the host bone (HB) – allograft (AB) overlap (Ov). **(B)** Hydroxyapatite (HA, red asterisks) was nearly fully incorporated by newly formed host bone, while osteoid seams (red arrows) were frequently apparent near host bone. **(C)** Glass ionomer cement (GI, red asterisks) presented with thick seams of unmineralized bone matrix (pink on the left, light blue on the right) as well as fibrosis near GI. **(D)** Overall, the bone volume fraction (*BV/TV*) was increased in the overlap region. **(E)** This was partly recapitulated by an elevated trabecular thickness (*Tb.Th*) compared to allograft bone. **(F)** Allograft bone, host bone, and overlap showed a similar trabecular number (*Tb.N*). **(G)** The amount of unmineralized bone matrix (osteoid) represented by the fraction of osteoid volume per bone volume (*OV/BV*) was elevated in the overlap region compared to allograft bone. Of note, AB with additional GI cement presented with an *OV/BV* over 40 %. **(H)** At the HB-AB overlap, a higher proportion of bone surface covered with osteoblasts (*Ob.S/BS*) was observed in comparison to AB and to HB. **(I)** Similarly, the overlap region presented a higher proportion of bone surface covered with osteoclasts (*Oc.S/BS*) compared to AB. **(J)** The number of vascular channels per marrow area (*N.VC/Ma.Ar*) was highest in the overlap region, specifically compared to allograft bone. However, even in the allograft bone region few blood vessels were apparent. **(K)** Similarly, the overlap region presented a higher proportion of vascular channel area per marrow area (*VC.Ar/Ma.Ar*) compared to HB and AB.

To further evaluate the osteoconductive capacity of allografts, micro-morphological and cellular analysis was performed on toluidine blue-stained and von Kossa-stained sections (Fig. 3A). The specimen with additional hydroxyapatite showed the most sclerotic bone structure at the interface region with well-incorporated hydroxyapatite fragments (Fig. 3B). The bone matrix adjacent to glass ionomer was mainly demineralized. Interestingly, the transition from unmineralized to mineralized bone also crossed individual bone lamellae suggesting active demineralization (Fig. 3C). The allograft-host bone overlap was characterized by a higher bone volume fraction compared to the host bone region (*p* = 0.0241) and allograft bone (*p* = 0.0339) (Fig. 3D). This was reflected by a higher trabecular thickness in the overlap region with a similar trabecular number between the three regions (Fig. 3E and F). In the presence of allograft and glass ionomer, the osteoid volume per bone volume was markedly high compared to the other cases. On average, the overlap presented with more osteoid than allograft bone (*p* = 0.003) (Fig. 3G) and with a higher proportion of bone surfaces covered with osteoblasts (*p*_*Ov-AB*_ < 0.0001, *p*_*Ov-HB*_ = 0.0027) and osteoclasts (*p*_*Ov-AB*_ = 0.0252) indicating activated bone remodelling (Fig. 3H and I). Analysis of the vascularisation of each region revealed that vascular channels were not only present in the host bone and the overlap region but even partly reached into the allograft bone. The overlap region was characterized by a higher number of vascular channels compared to allograft bone (*p* = 0.0167) and a higher vascular channel area within the marrow compared to both allograft bone (*p* = 0.0135) and the host bone region (*p* = 0.047) (Fig. 3J and K).

**Fig. 4:**
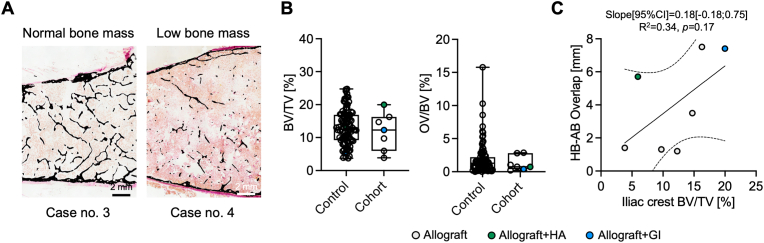
**Histomorphometric analysis of the overallskeletalstatus in trabecular bone of the iliac crest. (A)** Representative images of iliac crest sections stained with von Kossa/van Giesson indicating an age-related normal to low bone mass. **(B)** The bone volume fraction (*BV/TV*) and osteoid to bone volume fraction (*OV/BV*) were in the range of an age- and sex-matched control cohort [18]. (**C)** No significant association between iliac crest BV/TV and host bone-allograft bone overlap was apparent.

In addition to the bone remodelling activity at the interface of allograft and host bone, the overall bone status of the patients was assessed by analysing the trabecular bone of the iliac crest in each case. A range of normal to low bone mass was detected by histomorphometry on von Kossa-stained sections (Fig. 4A, Table S2). Compared to an age- and sex-matched control group, no difference was detected with respect to bone volume fraction (*p* = 0.543) or osteoid volume per bone volume (*p* = 0.724, Fig. 4B). No significant association was determined between the iliac bone volume fraction and the femoral allograft-host bone overlap (Fig. 4C).

### 3.3 Heterogenous and high mineralization of allograft bone

**Fig. 5:**
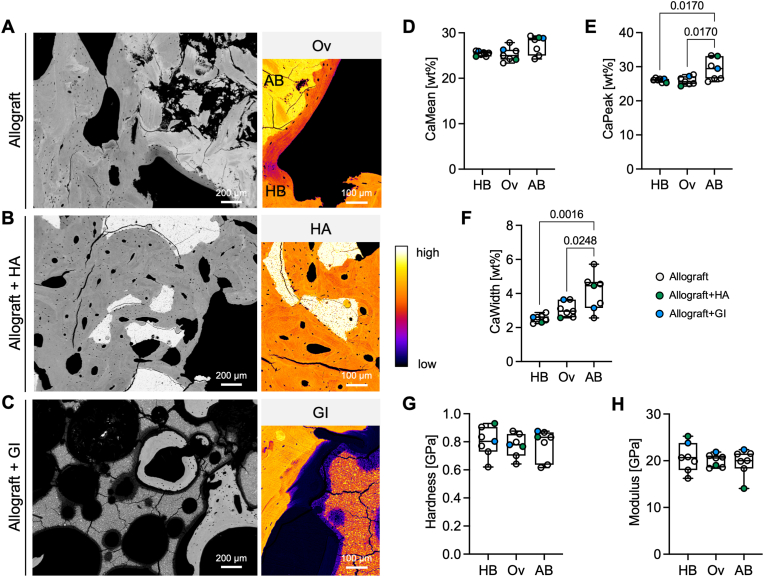
**Allograft bone presents with a higher degree of bone mineral content. (A)** Quantitative backscattered electron imaging (qBEI) was performed to determine the local bone mineral density distribution (BMDD), which is indicated by grey values representing the calcium content. In color-coded images of the overlap (Ov), fusion of host bone (HB) and highly mineralized allograft bone (AB) is apparent. **(B)** Hydroxyapatite (HA), which is characterized by a high calcium content, is completely incorporated and forms a phase contrast to host bone. **(C)** The contact zone of GI and bone was apparent with near absence of calcium in bone and a changed composition of GI compared to its central region. **(D)** By quantification of the calcium content, no change in the mean calcium content (*CaMean*) between the three region was detectable. **(E)** The peak calcium content (*CaPeak*) in AB was increased compared to HB and the overlap region. **(F)** Similarly, the mineralization heterogeneity (*CaWidth*) was elevated in AB. **(G,H)** Nanoindentation of the mineralized bone matrix in all regions was performed to analyze mechanical characteristics. HB, AB, and the overlap region did not present with differences in *Hardness* or *Modulus.*

To determine the quality of the bone matrix which is formed during allograft incorporation, the bone mineral density distribution and biomechanical characteristics were determined by qBEI and nanoindentation, respectively. In qBEI images, allograft and host bone were clearly distinguishable from each other, not only due to the fragmented structure of the allograft but also based on the high calcium content (Fig. 5A). Hydroxyapatite and the surrounding host bone matrix were homogenously composed of calcium (Fig. 5B). When evaluating bone with the presence of glass ionomer, an unmineralized gap to bone was apparent. Further, glass ionomer presented with a change in composition at its rim with a depth of approximately 25 μm (Fig. 5C). This observation was validated by energy dispersive x-ray absorptiometry (EDX) of interface regions. Herein, a compositional change from central regions to bone-near regions was observed indicating a release of alumina, fluoride, and silica, thus creating an acidic milieu with the potential of demineralizing surrounding bone (Figure S2). By quantification of the calcium content, no regional changes in mean calcium content (*CaMean*) between host bone, allograft, and overlap region were detectable (Fig. 5D). However, allograft bone presented with a higher *CaPeak* compared to both host bone (*p* = 0.017) and overlap (*p* = 0.017) (Fig. 5E). Furthermore, allograft bone presented with a more heterogeneous mineralization compared to host bone (*p* = 0.0016) and overlap (*p* = 0.0248) (Fig. 5F). Despite differences in matrix mineralization, no alterations in nanoindentation parameters, *i.e.*, hardness and modulus, were detected between the three regions, indicating preserved biomechanical properties across the whole interface (Fig. 5G and H).

### 3.4 The pronounced osteocyte lacuno-canalicular network in the overlap region is limited by the allograft-host bone interface

**Fig. 6:**
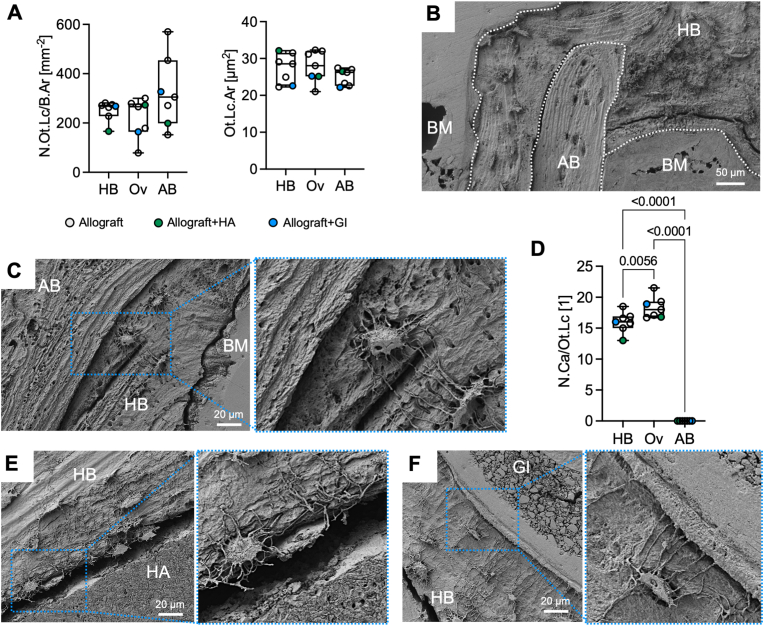
**The osteocyte lacuno-canalicular network (LCN) is limited by the allograft-host bone border. (A)** QBEI measurements were further used to identify osteocyte lacunae and determine the extent of the LCN. The number of osteocyte lacunae per bone area (*N.Ot.Lc/B.Ar*) as well as the mean osteocyte lacunar area (*Ot.Lc.Ar*) was similar in host bone (HB), allograft bone (AB) and overlap (Ov). **(B)** Additionally, acid etching and subsequent imaging with a scanning electron microscope was facilitated to depict the osteocyte lacunae and branching canaliculi. The interfaces of HB, AB, and bone marrow (BM) were clearly distinguishable. **(C)** Osteocyte lacunae were only found in HB, while AB was completely absent of lacunae. At the interface canaliculi attached to non-viable AB, but did not penetrate. **(D)** In the overlap region osteocyte lacunae presented with a higher number of canaliculi (*N.Ca/Ot.Lc*) compared to both other regions. **(E,F)** In samples with additional hydroxyapatite (HA) and glass ionomer (GI), osteocytes also arranged parallelly to its interface while canaliculi did also not penetrate the bone substitute materials.

QBEI images were further used to identify and quantify osteocyte lacunae within the mineralized bone matrix. In particular, the number of osteocyte lacunae (*N.Ot.Lc/B.Ar*) within the bone matrix was similar throughout the three regions. Although a trend towards smaller lacunae (*Ot.Lc.Ar*) in allograft bone was noticeable (*p* = 0.42), there were no significant differences in osteocyte lacunar size determined (Fig. 6A). To reveal the full extent of the osteocyte's lacuno-canalicular network (LCN), specimens were acid-etched, gold-sputtered, and imaged using a scanning electron microscope (Fig. 6B). Allograft bone was identified by the lack of osteocyte lacunae with branching canaliculi as well as a structural demarcation from host bone, which was frequently highlighted *via* a gap at the interface originating from the vacuum applied during scanning electron microscopy and which indicated a phase contrast between both tissues. Osteocytes aligned along this interface, while canaliculi touched but did not penetrate allograft bone (Fig. 6C). Further, the overlap region presented with a higher number of canaliculi per osteocyte lacuna compared to both host bone region (*p* = 0.0056) and allograft bone (*p* < 0.0001) assuming high interconnectivity and ability for communication between osteocytes and bone surface cells (Fig. 6D). Likewise, hydroxyapatite and glass ionomer showed an alignment of the osteocyte LCN along their interface to the host bone without penetration of canaliculi (Fig. 6E and F).

## 4 Discussion

Although the application of allografts and other bone substitute materials in femoral bone defects in the context of revision hip arthroplasty has proven to yield excellent outcomes in terms of clinical and radiological assessments, the incorporation process into host bone is still poorly understood. This study aimed to identify structural and cellular mechanisms as well as the factors driving and limiting allograft incorporation. For this purpose, a unique set of specimens from patients with successfully incorporated allografts was analysed on a microscopic, compositional, and cellular level.

Our results suggest that the incorporation of allograft bone in the proximal femur is primarily associated with the defect depth and may occur already shortly *(i.e.*, in the first months) after surgery, as highlighted by the lack of significant association between allograft time *in situ* and long-term incorporation. In line with this assumption, a previous study on the remodelling capacity of allografts in goats showed almost complete incorporation within the first 12 weeks after implantation [26]. Notably, we could not demonstrate a relationship between the overall bone status, as measured by the trabecular microstructure of the iliac crest, and allograft incorporation, indicating that allografts are also an option for patients with poor bone status (i.e., osteoporosis) to regenerate periprosthetic bone defects.

We demonstrated that bone cement penetration is one of the major limiting factors in this process, leading to inadequate remodelling of allograft bone and fibrosis development. A previous study found that fibrosis development on the acetabular side is dependent on the size of bone defect [9]. Although a direct association with incorporation indices was not apparent in this or previous studies, it was apparent that fibrotic tissue often encircled allograft bone separating it from host bone [8,9]. As indicated previously [11], bone cement penetration was associated with the presence of fibrosis. Hence, the osteoconductive properties of allograft bone are at least partially reduced by the presence of bone cement. The strong association between the defect depth and host bone-allograft overlap supports the hypothesis that a large defect reduces the penetration of bone cement up to the allograft-host bone interface and thus increases the vital contact area. Thus, our results suggest that in small defects, allograft use in combination with uncemented implant fixation may be used to increase the incorporation potential.

The osteoconductive capacity of allograft bone has been demonstrated in acetabular periprosthetic bone in the past [9,14,27,28]. In this study, the high remodelling rate, high vascularisation, and an extensive osteocyte LCN at the interface of host bone and allografts in femoral sections are clear indicators for the osteoconductive capacity of allograft bone leading to skeletal regeneration of periprosthetic bone loss [29]. Thereby, allograft bone was partly remodelled and incorporated, but not revitalized, which is highlighted by the clear demarcation of the interface between the vital osteocyte LCN in the host bone and the avital LCN in the allograft bone. However, the attachment of the osteocyte lacuno-canalicular network to allograft bone matrix indicates that osteocytes might act sensorially to locate avital bone and orchestrate its remodelling [30,31]. Besides indicating the current remodelling rate and bone quality, the extent of the osteocyte LCN is furthermore a mirror of past bone formation due to the transdifferentiation of osteoblasts into osteocytes, which might also suggest some osteoinductive potential of allografts [29,32]. Together, the factors responsible for the extent to which allograft bone is resorbed by osteoclasts and rebuilt by osteoblasts are not yet fully understood while emerging strategies for their manipulation provide the opportunity to enhance bone regeneration.

To address periprosthetic bone loss and provide additional stability for implant fixation during the revision surgery, allografts are supplemented by synthetic bone substitute materials in special cases, yielding good clinical outcomes [[33], [34], [35]]. Here, two specimens supplemented by hydroxyapatite and glass ionomer primarily enabled us to make a more precise assessment of allograft incorporation, especially the definition of individual allograft bone borders. Interestingly, we identified the highest osteoconductive capacity of allografts in combination with hydroxyapatite, which was completely incorporated (but not replaced) by host bone, indicating a synergistic effect of hydroxyapatite with allografts on osteoconduction. This finding is in line with radiological assessments of joint replacement surgeries with bulk hydroxyapatite and hydroxyapatite-coated implants as well as previous studies on its osteoconductive and biodegradable properties [15,[36], [37], [38]]. Although hydroxyapatite might inherit an even higher osteoconductive capacity compared to allograft bone, this observation might also indicate that the osseointegration of allograft bone is even more pronounced than apparent. In detail, during remodelling thick and highly mineralized bone substitute materials are slower degradable than thinner and less mineralized structures of avital bone [15,39]. In contrast to hydroxyapatite addition, glass ionomer showed a strong demineralizing capacity on host bone. Although the analysed case was successful in stabilizing the implant over 16 years, the effects on bone microstructure, mineralization, and cellular activation indicate that glass ionomer is not suitable as a bone substitute material for bone loss at major joints, *e.g.*, hip, knee, ankle, and shoulder. This observation indicates a rather antagonistic effect on allograft incorporation and is in line with the high failure rate reported in 45 cases in the early 1990s [40]. In the named study high concentrations of alumina in serum and bone marrow near implants were detected. Based on the current findings by EDX and qBEI measurements, alumina as well as silica, fluoride, and calcium are released from an approximately 25 μm thick layer from glass ionomer at the interface to bone.

This study has a few limitations. The relatively small sample size could be a reason for not being able to detect true differences between the three regions studied or associations with potential influencing factors. In particular, we were unable to provide a statistically meaningful analysis to support the regeneration potential in cases where synthetic bone substitutes (*e.g.* HA, GI) were added to the allografts. Nevertheless, it can be assumed that the defect depth is the major contributor to a successful osteoconduction and incorporation of allografts. Furthermore, this unique set of samples is the first to depict a holistic approach towards a better understanding of femoral allograft incorporation. Another limitation of our study is that we only included cases defined as 'clinically successful', and did not include failure cases. Hence, a more precise statement about individual causes of allograft failure cannot be made. However, our approach to include postmortem whole explants allowed us to decipher the full incorporation potential of allografts, which is usually not possible in clinical biopsy or retrieval studies. Finally, the histologically determined allograft bone border may not fully reflect the original defect depth. Therefore, the regenerative capacity of allografts through osteoconduction and subsequent remodelling may even be underestimated.

## 5 Conclusion

In this study, we have demonstrated the osteoconductive potential of bone allografts used during femoral revision arthroplasty for the treatment of periprosthetic bone defects. We provide clear evidence that allograft incorporation is accompanied by increased activity of bone cells responsible for degrading bone matrix and depositing new vital bone. The pronounced osteocyte LCN indicates a high cellular sensitivity to biomechanical stresses within the newly formed bone. Together, we show that regeneration of periprosthetic femoral bone loss is feasible through the use of allografts and provide the first scientific reference for their successful clinical application.

## Ethics approval statement

This study was approved by the local ethics committee (Ärztekammer Hamburg) under application WF-005/09 and complied with the Declaration of Helsinki. Informed consent was obtained from the next of kin.

## Data availability statement

The data that support the findings of this study are available from the corresponding author upon reasonable request.

## Funding

This study was funded by the Damp foundation under grant no. 2022–09 (to SvK, TR). The initial specimen collection was supported by the 10.13039/501100005986Stiftung Endoprothetik (to MA, KP and MH).

## Declaration of competing interest

All authors declare that there are no conflicts of interest in relation to this work.
