# Supplementary material for: Allografts promote skeletal regeneration of periprosthetic femoral bone loss
Source: J Orthop Translat. 2025 Apr 23;52:182–91. doi: 10.1016/j.jot.2025.04.004 (PMC12053977; doi:10.1016/j.jot.2025.04.004)
Supplement: Multimedia component 1 [file mmc1.docx]

Supplementary Material

Allografts promote skeletal regeneration of periprosthetic femoral bone loss

**Table S1.** Quantification of the host bone-allograft interface in each individual case.

| Case no. | Defect depth [mm] | Defect area [mm^2^] | Interface length [mm] | Mean overlap [mm] | Maximum overlap [mm] | Overlap of interface [%] |
| --- | --- | --- | --- | --- | --- | --- |
| 1 | 6.3 | 299.1 | 71.2 | 5.7 (2.7) | 11.3 | 100 |
| 2 | 2.7 | 53.4 | 78.9 | 1.2 (0.4) | 2.6 | 100 |
| 3 | 10.2 | 369.8 | 66.7 | 7.4 (4.1) | 15.8 | 90.7 |
| 4 | 2.4 | 32.7 | 83.4 | 1.4 (1.8) | 7.5 | 100 |
| 5 | 4.7 | 102.8 | 16.8 | 3.5 (1.2) | 5.5 | 100 |
| 6 | 1.9 | 33.6 | 55.7 | 1.3 (0.6) | 2.5 | 95.2 |
| 7 | 8.2 | 48.0 | 14.6 | 7.5 (2.0) | 9.1 | 100 |

**Table S2.** Histomorphometric evaluation of iliac crest biopsies to assess the overall bone status.

| Case no. | BV/TV  [%] | Tb.Th  [µm] | Tb.N  [mm^-1^] | OV/BV  [%] | OS/BS  [%] | O.Th  [µm] |
| --- | --- | --- | --- | --- | --- | --- |
| 1 | 5.95 | 64.0 | 0.93 | 0.74 | 6.56 | 3.89 |
| 2 | 12.29 | 82.1 | 1.50 | 0.38 | 3.83 | 3.62 |
| 3 | 10.2 | 369.8 | 66.7 | 7.4 ± 4.1 | 15.8 | 90.7 |
| 4 | 2.4 | 32.7 | 83.4 | 1.4 ± 1.8 | 7.5 | 100 |
| 5 | 4.7 | 102.8 | 16.8 | 3.5 ± 1.2 | 5.5 | 100 |
| 6 | 1.9 | 33.6 | 55.7 | 1.3 ± 0.6 | 2.5 | 95.2 |
| 7 | 8.2 | 48.0 | 14.6 | 7.5 ± 2.0 | 9.1 | 100 |

BV/TV: bone volume per total volume, Tb.Th: trabecular thickness, Tb.N: trabecular number, OV/BV: osteoid volume per bone volume, OS/BS: osteoid surface per bone surface, O.Th: osteoid thickness.

**Figure S1. Fibrosis development at the interface of synthetic bone substitute materials and allograft bone. (A)** Representative image of a toluidine blue-stained ground section illustrating the allograft-allograft interface with fibrotic tissue (FT) development surrounding allograft bone (AB). **(B)** Representative image of a ground section showing the hydroxyapatite (HA) – allograft bone interface with a fibrous tissue layer separating both grafts. **(C)** Representative image of a toluidine blue-stained ground section of the glass ionomer (GI) – allograft bone interface with a fibrous tissue layer separating both grafts. A direct interface of both synthetic bone substitute materials with allograft was not apparent in any analysed section.

**Figure S2. Energy dispersive x-ray absorptiometry (EDX) of interfaces between host bone and additional bone substitute materials. (A)** Left panel: Colour-coded image of the hydroxyapatite–bone interface indicating a similar composition of bone materials in relation to calcium and phosphate. Right panel: Representative mean calcium content and calcium to phosphate ratio in the labelled areas. **(B)** Left panel: Colour-coded image of the glass ionomer (GI)–bone interface presenting a compositional change of the GI near bone to less alumina, silica, and fluoride. Right panel: Low calcium content in bone near GI indicating a demineralization of bone. Quantification of GI composition indicated dissolved calcium, alumina, silica, and fluoride in a layer of approximately 25 µm at its interface to bone.
